# Influence of maternal diabetes during pregnancy on ultrasound-measured fetal epicardial fat thickness: A meta-analysis

**DOI:** 10.17305/bb.2025.11909

**Published:** 2025-03-05

**Authors:** Apizi Anwaier, Jian Li, Wei Liu, Liangjie Dong, Yunfei Ding, Zhaoxia Yu

**Affiliations:** 1 Department of Critical Care Medicine, The First affiliated Hospital of Xinjiang Medical University, Urumqi, China

**Keywords:** Gestational diabetes mellitus, GDM, pregestational diabetes mellitus, PDM, fetal epicardial fat thickness, fEFT, metabolism, meta-analysis

## Abstract

Maternal diabetes during pregnancy, including gestational diabetes mellitus (GDM) and pregestational diabetes mellitus (PDM), has been linked to alterations in fetal development. This meta-analysis aimed to investigate the impact of maternal diabetes on fetal epicardial fat thickness (fEFT), measured via ultrasound—a potential marker of cardiometabolic risk. A systematic search of PubMed, Embase, and Web of Science was conducted to identify observational studies assessing fEFT in pregnant women with and without diabetes. A random-effects model was used to calculate the mean difference (MD) in fEFT between groups. Heterogeneity was evaluated using the *I*^2^ statistic, and sensitivity, subgroup, and meta-regression analyses were performed to explore sources of variability. Data from 10 studies, comprising 12 datasets and 1303 participants, were pooled. Women with diabetes during pregnancy had significantly higher fEFT compared to those without diabetes (MD: 0.37 mm, 95% confidence interval [CI]: 0.26 to 0.49, *P* < 0.001), with moderate heterogeneity (*I*^2^ ═ 69%). Sensitivity analyses, conducted by excluding one dataset at a time, confirmed the robustness of the findings (all *P* values < 0.05). Meta-regression revealed a positive correlation between gestational age (GA) at fEFT measurement and fEFT differences (coefficient ═ 0.040, *P* ═ 0.005), accounting for 83.2% of the heterogeneity. Subgroup analyses demonstrated consistent results across study designs, maternal diabetes types, and demographic factors but highlighted greater fEFT differences in studies where GA at fEFT measurement was >26 weeks. In conclusion, maternal diabetes during pregnancy is associated with increased fEFT, particularly in later gestation.

## Introduction

Diabetes mellitus (DM) is a common metabolic disorder with significant global health implications, particularly among women of childbearing age [1, 2]. Gestational diabetes mellitus (GDM), defined as glucose intolerance first recognized during pregnancy [3], and pregestational diabetes mellitus (PDM), which includes type 1 or type 2 diabetes diagnosed before pregnancy [4], affect a substantial number of pregnancies worldwide. GDM alone is estimated to complicate approximately 14% of pregnancies [5], while PDM incidence varies by region, reflecting broader diabetes prevalence trends [6]. Both conditions are associated with adverse maternal and fetal outcomes, including preeclampsia, preterm delivery, fetal macrosomia, and perinatal complications [7–9]. These risks highlight the need to better understand and mitigate the impact of maternal DM on pregnancy and offspring health. Emerging evidence suggests that maternal DM may influence the cardiometabolic health of offspring, both in utero and later in life [10, 11]. Fetal exposure to maternal hyperglycemia is thought to disrupt metabolic programming, increasing the risk of obesity, insulin resistance, and type 2 diabetes in adolescence and adulthood [12]. This has led to growing interest in identifying early markers of cardiometabolic risk in fetuses exposed to maternal DM [13]. Among these, fetal epicardial fat thickness (fEFT) has emerged as a promising candidate. Epicardial fat, a metabolically active visceral fat depot surrounding the myocardium and coronary arteries, is linked to cardiometabolic disorders in adults [14, 15]. Measuring fEFT via ultrasound provides a non-invasive method to assess fetal adiposity and may offer insights into early metabolic alterations influenced by maternal factors [16]. Increased fEFT in fetuses and neonates correlates with higher birth weight, greater adiposity, and early metabolic dysfunction markers, such as hyperinsulinemia [17–20]. These findings suggest that elevated fEFT in utero could serve as an early indicator of cardiometabolic risk. Despite the biological plausibility and clinical significance of these associations, research on the effects of maternal DM on fEFT remains limited. Some observational studies have reported increased fEFT in fetuses of women with GDM or PDM compared to non-diabetic pregnancies [21–28]. However, findings across studies have been inconsistent due to differences in study design, measurement techniques, and sample characteristics. Given the growing interest in fEFT as a potential early marker of fetal cardiometabolic risk, understanding its association with maternal diabetes is crucial. While individual studies have explored this relationship, their findings vary. To address this, we conducted a systematic review and meta-analysis to quantitatively assess the impact of maternal diabetes (both GDM and PDM) on ultrasound-measured fEFT. Additionally, we examined potential sources of heterogeneity, including gestational age (GA) at fEFT measurement, maternal BMI, and the type of maternal DM, to provide a comprehensive synthesis of the available evidence.

## Materials and methods

This meta-analysis followed the guidelines of the Preferred Reporting Items for Systematic Reviews and Meta-Analyses (PRISMA 2020) [29, 30] and the Cochrane Handbook for Systematic Reviews of Interventions [31] in its design, data collection, statistical analysis, and interpretation of results. It was registered with PROSPERO under the identifier CRD42024618929. Originally, the protocol focused on pregnancies complicated by GDM compared to controls. However, before data extraction, it was amended to include both GDM and PDM to provide a more comprehensive evaluation of maternal diabetes’ impact on fetal epicardial fat thickness. This amendment was submitted to and approved by PROSPERO in accordance with standard meta-analysis procedures.

### Literature search

To identify studies relevant to the meta-analysis, we conducted a systematic search of PubMed, Embase, and Web of Science using comprehensive search terms. These included (“epicardial adipose tissue” OR “epicardial fat” OR “pericardial adipose tissue” OR “pericardial fat” OR “cardiac adipose tissue” OR “cardiac fat” OR “subepicardial adipose tissue” OR “subepicardial fat” OR “heart fat” OR “heart adipose tissue”) AND (“gestational diabetes” OR “GDM” OR “pregestational diabetes” OR “gestational” OR “pregnancy” OR “pregnant”) AND (“diabetes” OR “diabetic” OR “hyperglycemia”). The search was limited to studies involving humans and published as full-length articles in peer-reviewed English-language journals. Additionally, we manually screened references from relevant original and review articles to identify any additional eligible studies. The literature search covered publications from the inception of each database through November 12, 2024. Detailed search terms and strategies for each database are provided in the Supplemental Data.

### Inclusion and exclusion criteria

The inclusion criteria for eligible studies were as follows: (1) observational studies published as full-length articles; (2) studies including pregnant women with DM—either GDM or PDM—as well as healthy pregnant women without DM, all with singleton pregnancies; (3) studies that assessed fEFT via ultrasound in women with and without DM; and (4) studies that reported or allowed for the calculation of differences in fEFT between women with and without DM during pregnancy. The diagnostic criteria for GDM or PDM were based on those used in the included studies. The exclusion criteria were as follows: (1) studies that did not include pregnant women; (2) studies that included pregnant women with other clinical conditions, such as pregnancy-induced hypertension or preeclampsia; (3) studies that did not measure fEFT; and (4) preclinical studies, reviews, or editorials. In cases of overlapping populations, the study with the largest sample size was included in the meta-analysis.

### Study quality evaluation and data extraction

The literature search, study identification, quality assessment, and data extraction were independently conducted by two authors. Any disagreements were resolved through consultation with the corresponding author. Study quality was assessed using the Newcastle-Ottawa Scale (NOS) [32], which evaluates three domains: selection of cases and controls, comparability between groups, and measurement of exposure. The NOS assigns scores from 1 to 9, with higher scores indicating better quality. The following data were extracted from each study for analysis: study details (author, year, country, and design), participant characteristics (sample size, age, and BMI of pregnant women), median GA for fEFT measurement, type of maternal DM (GDM or PDM), and variables matched or adjusted when assessing the influence of maternal DM on fEFT.

### Statistical analysis

The mean difference (MD) with corresponding 95% confidence intervals (CIs) was used to summarize the difference in fEFT between pregnant women with and without diabetes [33]. Heterogeneity among studies was assessed using the Cochrane *Q* test and the *I*^2^ statistic [33, 34] and categorized as mild (*I*^2^ < 25%), moderate (*I*^2^ 25%–75%), or substantial (*I*^2^ > 75%). A random-effects model was applied to pool the results, accounting for potential between-study variability [31]. Sensitivity analyses were conducted by omitting one dataset at a time to evaluate the robustness of the findings [33]. Predefined univariate meta-regression analyses were performed to assess the modifying effects of study characteristics on the outcomes, including sample size, mean maternal age, mean BMI, and NOS scores. Additionally, predefined subgroup analyses explored the influence of study characteristics, such as study design, type of maternal diabetes, mean maternal age, BMI, timing of fEFT measurement, and NOS scores, using medians of continuous variables as cutoffs for subgroup definitions. Publication bias was initially assessed through funnel plot construction and visual examination of symmetry [35], complemented by Egger’s regression test 35. Statistical analyses were performed using RevMan (Version 5.1; Cochrane Collaboration, Oxford, UK) and Stata software (Version 12.0; Stata Corporation, College Station, TX, USA). A two-sided *P* value of <0.05 was considered statistically significant.

## Results

### Study inclusion

**Figure 1.**
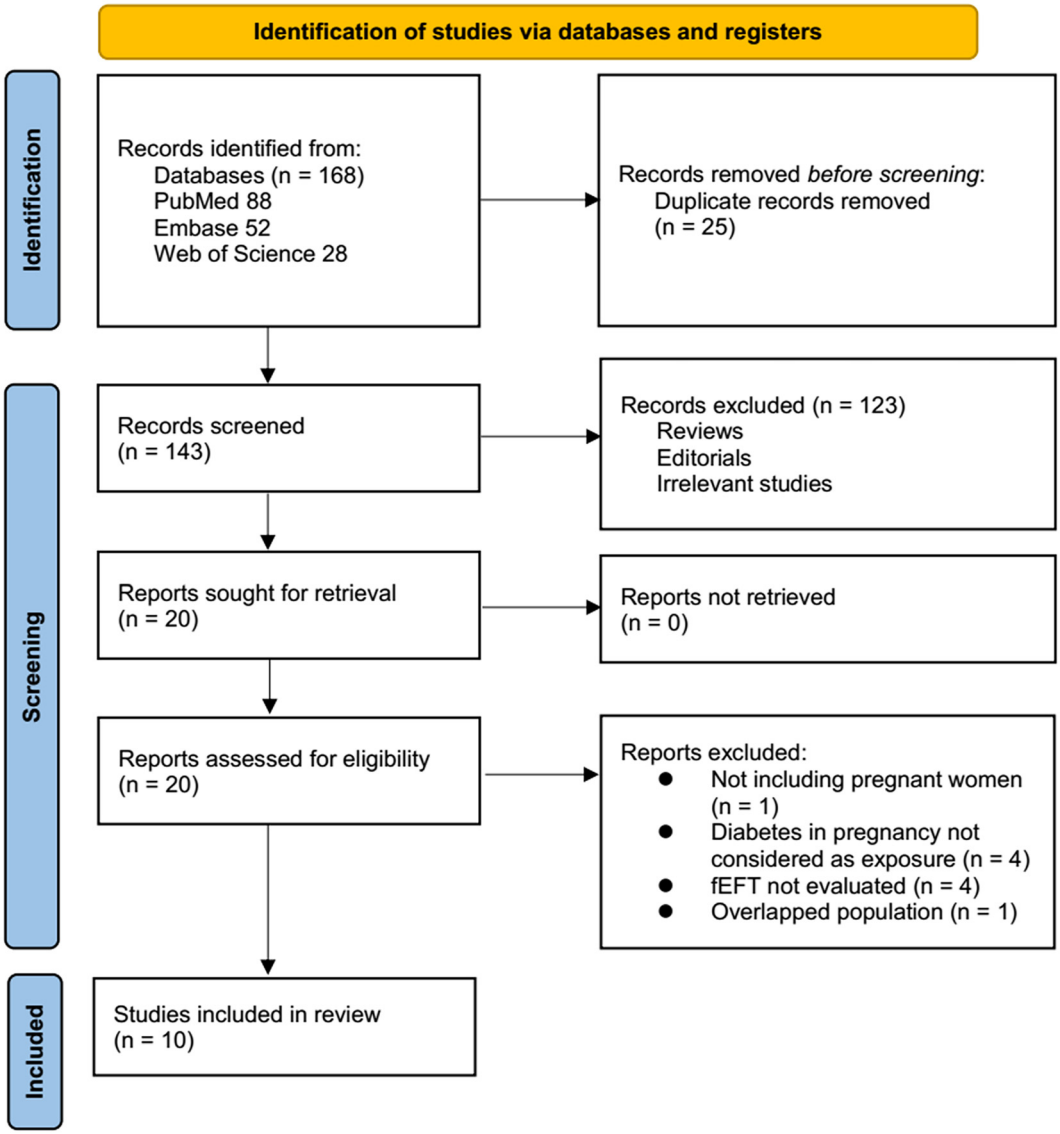
Flowchart illustrates the process of database search and study identification.

The study inclusion process is illustrated in Figure 1. A comprehensive search of three databases initially identified 168 potentially relevant records. After removing 25 duplicates, 143 records remained. Title and abstract screening excluded 123 studies, primarily due to misalignment with the meta-analysis objectives. The full texts of the remaining 20 records were then independently assessed by two authors, resulting in the exclusion of 10 studies for reasons detailed in Figure 1. Ultimately, 10 observational studies were deemed eligible for inclusion in the quantitative analysis [21–28, 36, 37].

**Table 1:** Characteristics of the included studies

**Study**	**Country**	**Design**	**No. of women included**	**Maternal age (years)**	**Maternal BMI (kg/m^2^)**	**fEFT measuring timing**	**Methods for fEFT measuring**	**Median GA of fEFT measuring (weeks)**	**Type of maternal diabetes**	**No. of women with DM**	**Variables matched or adjusted**
Yavuz, 2016	Türkiye	CS	80	27.7	27.9	GA: 24–28 weeks	Via ultrasound at end-diastole over 3 cardiac cycles from the RV wall, recording the highest value per cycle	26	GDM (IADPSG)	40	Maternal age, BMI, GA, fetal gender, and fetal abdominal circumference
Jackson, 2016	USA	CS	56	28.8	30.8	GA: 20–28 weeks	Via ultrasound using LVOT views near the aortic valve, selecting the image that best delineated the epicardial fat border	23.5	PDM	28	Maternal age, BMI, GA, and fetal abdominal circumference
Akkurt, 2018	USA	CC	212	29.2	31.3	GA: 24–35 weeks	Via ultrasound using the LVOT view, identifying the hypoechogenic fat layer over the RV, and measuring the thickest point near a reference line drawn through the descending aorta and aortic annulus	28.2	GDM (IADPSG)	106	Maternal BMI, parity, ethnicity, GA, fetal sex, and fetal abdominal circumference
Aydin, 2020	Türkiye	CC	120	32.1	29	GA: 18–22 weeks	Via ultrasound using the standardized four-chamber view, measuring fEFT at the midpoint of the ventricular wall	20	GDM (IADPSG)	60	Maternal age, BMI, GA, and fetal abdominal circumference
Iskender, 2022	Türkiye	CC	80	28	29.1	GA: 28–39 weeks	Via ultrasound using the apical five-chamber view, measuring the hypoechoic area on the free wall of the RV with a reference line drawn from the descending aorta through the aortic annulus	34.5	GDM (IADPSG)	40	Maternal age, gravidity, and GA
Baria, 2023	India	CS	70	35.8	NR	GA: 24–28 weeks	Via ultrasound using the LVOT view, identifying the hypoechogenic area between the visceral pericardium and myocardium along the right ventricle, measured during end-systole	26	GDM (DIPSI)	35	Maternal age and GA
Ghuman, 2023	India	CC	70	25.6	NR	GA: 24–32 weeks	Via ultrasound using a standardized four-chamber view at end-diastole over three cardiac cycles, measuring the hypoechoic space just outside the myocardium on the right ventricular free wall	28	GDM (IADPSG)	35	Maternal age, BMI, and GA
Omeroglu, 2023 GDM	Türkiye	CC	135	29	30.4	GA: 28–39 weeks	Via ultrasound using the LVOT view, measuring the hypoechoic area between the myocardium and visceral pericardium on the RV	32	GDM (IADPSG)	90	GA
Omeroglu, 2023 PDM	Türkiye	CC	90	28	29.9	GA: 28–39 weeks	Via ultrasound using the LVOT view, measuring the hypoechoic area between the myocardium and visceral pericardium on the RV	32	PDM	45	GA
Singh, 2023	India	CS	60	26.4	NR	GA: 24–28 weeks	Via ultrasound using the LVOT view, measuring the hypoechoic area between the visceral pericardium and myocardium along the right ventricle at end-diastole	26	GDM (IADPSG)	30	Maternal age and GA
Sever, 2023 GDM	Türkiye	CC	165	29.9	30	GA: 24–28 weeks	Via ultrasound using cardiac long-axis views of the LVOT near the aortic valve, measuring the thickest hypoechoic area at end-diastole	26	GDM (IADPSG)	110	Maternal BMI, GA, parity, gravidity, and fetal sex
Sever, 2023 PDM	Türkiye	CC	165	30.6	30.1	GA: 24–28 weeks	Via ultrasound using cardiac long-axis views of the LVOT near the aortic valve, measuring the thickest hypoechoic area at end-diastole	26	PDM	110	Maternal BMI, GA, parity, gravidity, and fetal sex

GDM: Gestational diabetes mellitus; PDM: Pregestational diabetes mellitus; GA: Gestational age; fEFT: Fetal epicardial fat thickness; DM: Diabetes mellitus.

### Summary of study characteristics

Table 1 summarizes the characteristics of the included studies. In total, 10 observational studies were analyzed, consisting of six case-control studies [23, 24, 26–28, 36] and four cross-sectional studies [21, 22, 25, 37]. These studies, published between 2016 and 2023, were conducted in Türkiye [22, 24, 27, 28, 36], the United States [21, 23], and India [25, 26, 37]. Two studies [27, 28] provided separate datasets for women with GDM and PDM, resulting in a total of 12 datasets included in the meta-analysis. Across all studies, 1303 women with singleton pregnancies were analyzed, with mean maternal ages ranging from 25.6 to 35.8 years and mean BMIs from 27.9 to 31.3 kg/m^2^. The ultrasonic methods for measuring fEFT varied, including left ventricular outflow tract, four-chamber, and apical views, with different reference points for defining fEFT thickness—details of which are provided in Table 1. The median GA at ultrasound assessment of fEFT ranged from 20.0 to 34.5 weeks. Among the studies, six included women with GDM [22, 24–26, 36, 37], one focused on women with PDM [21], and three included both GDM and PDM populations [23, 27, 28]. In all studies, potential confounding factors—such as GA at the time of fEFT measurement—were matched between women with and without diabetes during pregnancy. The methodological quality of the included studies, assessed using the NOS, ranged from seven to nine stars, indicating an overall good quality of evidence (Table 2).

**Table 2:** Study quality evaluation via the Newcastle-Ottawa Scale

	**Adequate definition of the cases**	**Representativeness of the cases**	**Selection of controls**	**Definition of controls**	**Controlled for GA**	**Controlled for other confoundings**	**Ascertainment of the exposure**	**Same method of ascertainment of exposure for cases and controls**	**Non-response rate**	**Overall**
Yavuz, 2016	1	0	1	1	1	1	1	1	1	8
Jackson, 2016	1	1	1	1	1	1	1	1	1	9
Akkurt, 2018	0	0	1	1	1	1	1	1	1	7
Aydin, 2020	1	1	1	1	1	1	1	1	1	9
Iskender, 2022	1	1	1	1	1	1	1	1	1	9
Baria, 2023	1	0	1	1	1	1	1	1	1	8
Ghuman, 2023	1	0	1	1	1	1	1	1	1	8
Omeroglu, 2023 GDM	1	0	1	1	1	0	1	1	1	7
Omeroglu, 2023 PDM	1	0	1	1	1	0	1	1	1	7
Singh, 2023	1	0	1	1	1	1	1	1	1	8
Sever, 2023 GDM	1	0	1	1	1	1	1	1	1	8
Sever, 2023 PDM	1	0	1	1	1	1	1	1	1	8

GDM: Gestational diabetes mellitus; PDM: Pregestational diabetes mellitus; GA: Gestational age.

### Results of overall meta-analysis and sensitivity analysis

**Figure 2.**
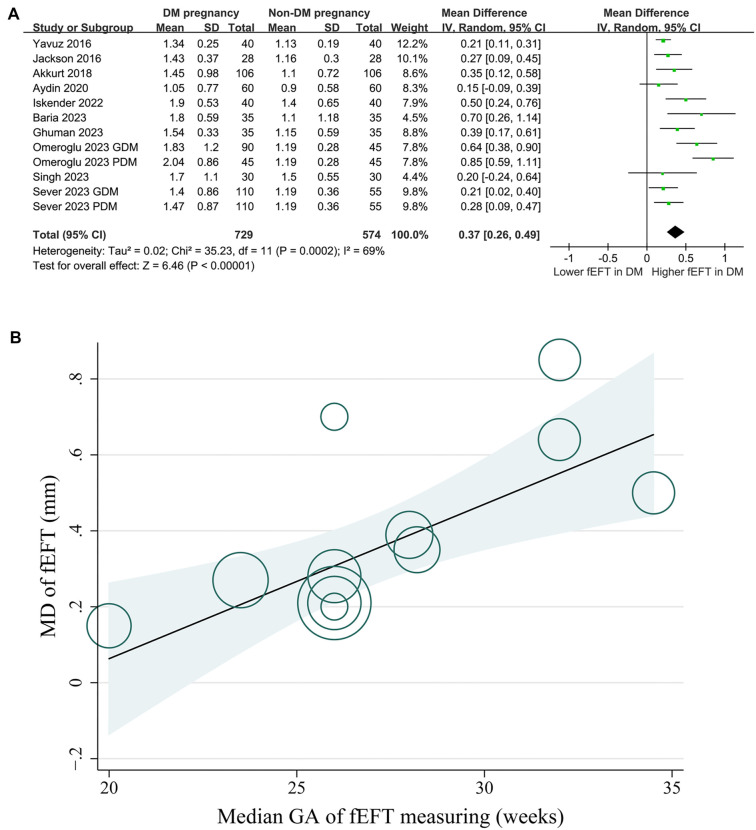
**Forest plots for the meta-analysis comparing fEFT between women with and without DM in pregnancy and plots of the meta-regression analysis for the influence of GA of fEFT assessment.** (A) Forest plots for the overall meta-analysis and (B) Meta-regression for the influence of GA of fEFT assessment on the results. GDM: Gestational diabetes mellitus; PDM: Pregestational diabetes mellitus; GA: Gestational age; fEFT: Fetal epicardial fat thickness; DM: Diabetes mellitus; CI: Confidence interval; MD: Mean difference.

Moderate heterogeneity was observed among the included studies (*I*^2^ ═ 69%). Using a random-effects model, the pooled analysis showed that fEFT was significantly higher in women with DM during pregnancy compared to those without DM (MD: 0.37 mm, 95% CI: 0.26–0.49, *P* < 0.001; Figure 2A). Sensitivity analyses, conducted by excluding one dataset at a time, confirmed the robustness of the results (MD range: 0.32–0.40, all *P* < 0.05).

**Table 3:** Results of univariate meta-regression analysis

**Variables**	**MD of fEFT between women with and without DM in pregnancy**			
	**Coefficient**	**95% CI**	***P* values**	**Adjusted *R*^2^**
Sample size	−0.00061	−0.00360 to 0.00237	0.65	−14.9%
Mean maternal age (years)	0.0035	−0.0614 to 0.0684	0.91	−14.5%
Mean maternal BMI (kg/m^2^)	0.041	−0.111 to 0.194	0.56	−10.8%
Median GA at fEFT measuring (weeks)	0.040	0.015 to 0.066	0.005	83.2%
NOS	−0.15	−0.32 to 0.03	0.09	28.7%

GA: Gestational age; fEFT: Fetal epicardial fat thickness; DM: Diabetes mellitus; CI: Confidence interval; MD: Mean difference; NOS: Newcastle-Ottawa Scale.

### Results of the meta-regression analysis

Univariate meta-regression analysis revealed a positive correlation between the median GA at fEFT measurement and the fEFT difference between women with and without DM during pregnancy (coefficient ═ 0.040, *P* ═ 0.005; Table 3, Figure 2B), accounting for a substantial proportion of heterogeneity (Adjusted *R*^2^ ═ 83.2%). Other variables, including sample size, mean maternal age, mean maternal BMI, and NOS scores, did not show significant effects (all *P* > 0.05; Table 3).

### Results of the subgroup analysis

**Figure 3.**
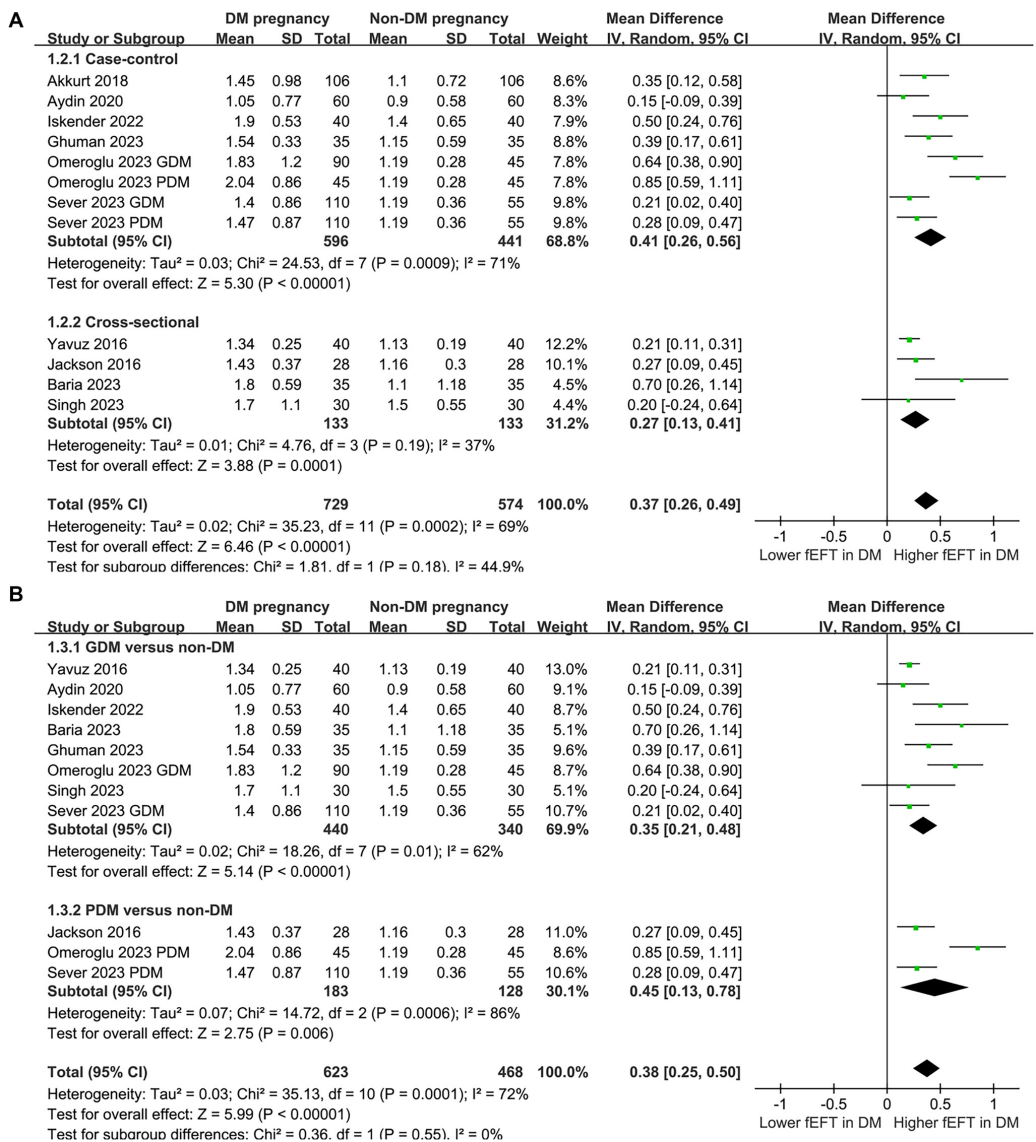
**Forest plots for the subgroup analyses comparing fEFT between women with and without DM in pregnancy.** (A) Subgroup analysis according to study design and (B) Subgroup analysis according to the type of DM in pregnancy. GDM: Gestational diabetes mellitus; PDM: Pregestational diabetes mellitus; GA: Gestational age; fEFT: Fetal epicardial fat thickness; DM: Diabetes mellitus; CI: Confidence interval; MD: Mean difference.

**Figure 4.**
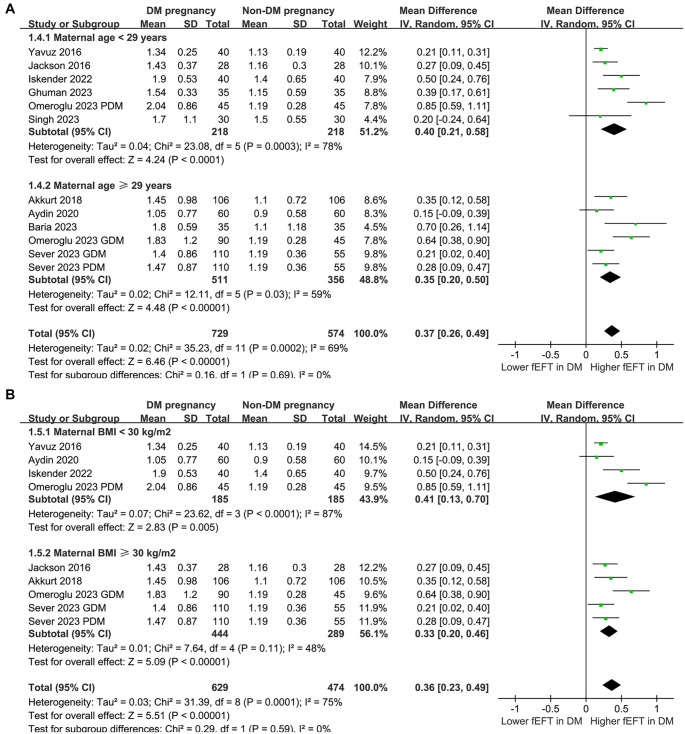
**Forest plots for the subgroup analyses comparing fEFT between women with and without DM in pregnancy.** (A) Subgroup analysis according to the mean maternal age and (B) Subgroup analysis according to the mean maternal BMI. GDM: Gestational diabetes mellitus; PDM: Pregestational diabetes mellitus; GA: Gestational age; fEFT: Fetal epicardial fat thickness; DM: Diabetes mellitus; CI: Confidence interval; MD: Mean difference.

**Figure 5.**
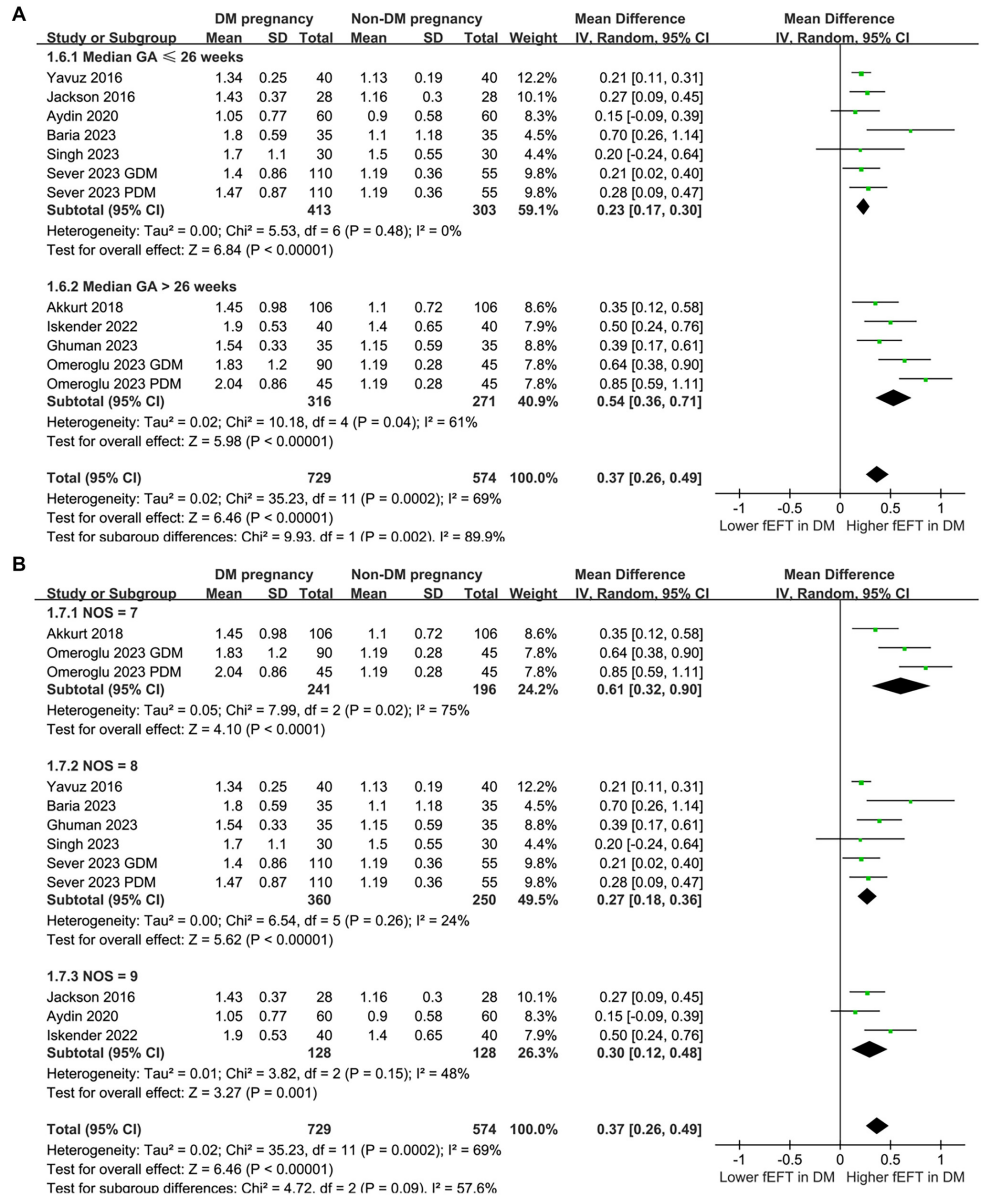
**Forest plots for the subgroup analyses comparing fEFT between women with and without DM in pregnancy.** (A) Subgroup analysis according to the median GA of fEFT measurement and (B) Subgroup analysis according to the NOS scores. GDM: Gestational diabetes mellitus; PDM: Pregestational diabetes mellitus; GA: Gestational age; fEFT: Fetal epicardial fat thickness; DM: Diabetes mellitus; CI: Confidence interval; MD: Mean difference; NOS: Newcastle-Ottawa Scale.

Subgroup analyses revealed consistent effects of maternal DM on fEFT across study designs (case-control and cross-sectional; *P* for subgroup difference ═ 0.18; Figure 3A), maternal DM types (GDM and PDM; *P* for subgroup difference ═ 0.55; Figure 3B), maternal age categories (< 29 vs ≥ 29 years; *P* ═ 0.69; Figure 4A), and maternal BMI categories (< 30 vs ≥ 30 kg/m^2^; *P* ═ 0.59; Figure 4B). However, subgroup analysis by GA for fEFT measurement showed a significantly greater increase in fEFT in studies with GA > 26 weeks compared to those with GA ≤ 26 weeks (0.54 vs 0.23 mm; *P* for subgroup difference ═ 0.002; Figure 5A). A similar trend was observed in studies with varying NOS scores (*P* for subgroup difference ═ 0.09; Figure 5B).

### Publication bias

**Figure 6.**
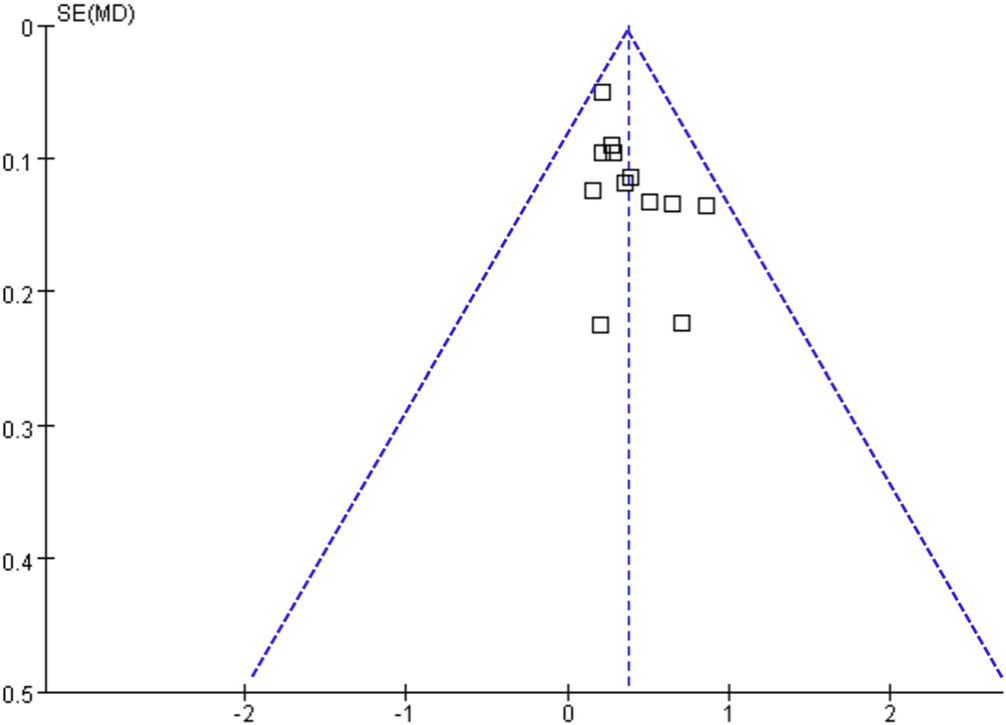
**Funnel plots for evaluating the possible publication bias of the meta-analysis comparing fEFT between women with and without DM in pregnancy.** fEFT: Fetal epicardial fat thickness; DM: Diabetes mellitus; MD: Mean difference.

Figure 6 presents the funnel plots for the meta-analysis assessing the difference in fEFT between women with and without DM during pregnancy. The plots appear symmetrical upon visual inspection, suggesting a low risk of publication bias. This observation is further supported by Egger’s regression test, which did not indicate significant publication bias (*P* ═ 0.58).

## Discussion

The results of this meta-analysis reveal a significant association between maternal DM during pregnancy and increased fEFT, with an MD of 0.37 mm compared to pregnancies without DM. This finding remained consistent across sensitivity analyses, with moderate heterogeneity observed. Meta-regression analysis identified GA at the time of fEFT measurement as a significant source of heterogeneity, suggesting that the impact of maternal DM on fEFT becomes more pronounced as pregnancy progresses. Subgroup analyses further confirmed this association across various study designs, maternal demographic factors, and study quality, underscoring the robustness of the findings. The influence of maternal DM on fEFT can be attributed to several pathophysiological mechanisms. Hyperglycemia-induced fetal hyperinsulinemia plays a central role, as elevated insulin levels stimulate adipocyte proliferation and hypertrophy, leading to increased fat deposition [38]. Insulin, a potent growth-promoting hormone during fetal development, directly influences the differentiation of preadipocytes into mature adipocytes, particularly in metabolically active depots such as epicardial fat [39]. Due to its proximity to the myocardium and coronary arteries, epicardial fat exhibits high lipolytic activity and secretes pro-inflammatory cytokines and adipokines [40], making it especially susceptible to metabolic alterations associated with maternal DM. Additionally, maternal DM is linked to systemic inflammation and oxidative stress, which may exacerbate adipogenesis and disrupt normal fat distribution in the fetus [41]. Hyperglycemia triggers excessive reactive oxygen species (ROS) production [42] and activates pro-inflammatory pathways such as nuclear factor-κB (NF-κB) [43]. This leads to the upregulation of inflammatory cytokines, including tumor necrosis factor-α (TNF-α) and interleukin-6 (IL-6), which further promote adipose tissue expansion and dysfunction [44]. In the fetal environment, these inflammatory signals may enhance epicardial fat deposition by stimulating local adipocyte proliferation and hypertrophy [45]. Maternal DM also affects placental function, further contributing to increased fEFT [46]. The placenta mediates nutrient transfer and endocrine signaling between the mother and fetus [47]. In pregnancies complicated by DM, placental abnormalities—including increased vascular resistance and reduced mitochondrial function—have been observed [47]. These changes can alter fetal glucose and lipid supply, favoring excessive energy availability and fat deposition [47]. Moreover, maternal hyperglycemia upregulates placental glucose and fatty acid transporters, increasing the flux of these substrates to the fetus and promoting adipogenesis in depots such as epicardial fat [48]. Finally, epigenetic modifications may also play a role in the impact of maternal DM on fEFT. Chronic hyperglycemia during pregnancy can induce changes in DNA methylation, histone modification, and non-coding RNA expression in the developing fetus [49]. These alterations can affect genes involved in adipogenesis and metabolism, potentially predisposing the fetus to increased fat deposition and cardiometabolic dysfunction later in life [50]. Studies have identified altered methylation patterns in genes regulating insulin signaling and lipid metabolism in offspring of diabetic pregnancies, which may contribute to increased fEFT [51, 52].

The results of the meta-regression and subgroup analyses provide key insights into the timing and magnitude of maternal DM’s effects on fEFT. The positive correlation between GA and fEFT differences suggests that later gestation may be a critical period for maternal DM’s influence on fetal adiposity. This finding has clinical implications, emphasizing the importance of early and sustained glycemic control throughout pregnancy to minimize its impact on fetal development [53]. The consistency of results across study designs, maternal age, BMI, and study quality indicates that the observed association is robust and unlikely to be confounded by these factors. This reinforces the validity of fEFT as a marker for assessing maternal DM’s effects on fetal development. Although our subgroup analysis did not reveal a significant difference in the effects of GDM vs PDM on fEFT, we recognize that these conditions have distinct metabolic characteristics and may influence fetal development differently [54]. However, their shared pathophysiological pathways—such as hyperglycemia-induced fetal hyperinsulinemia and inflammatory processes—likely contribute to similar changes in fEFT [55]. Further research is needed to explore potential variations in fEFT progression between GDM and PDM pregnancies, particularly in relation to glycemic control and disease severity. This meta-analysis has several strengths. It provides a comprehensive and up-to-date synthesis of the literature, incorporating data from multiple observational studies that match or adjust for GA to ensure comparability. Rigorous methodological approaches, including sensitivity, subgroup, and meta-regression analyses, were employed to explore heterogeneity and identify key modifiers of the observed association [56]. These analyses enhance the reliability of the findings and offer valuable insights into the factors influencing fEFT in pregnancies complicated by DM. Moreover, the inclusion of studies with high methodological quality, as assessed by NOS scores, further strengthens the robustness of the results. Despite these strengths, several limitations must be acknowledged. First, the meta-analysis includes only observational studies, which are inherently subject to residual confounding despite adjustments for key variables [57]. Second, unmeasured factors, such as maternal diet, physical activity, and genetic predispositions may have influenced the results [58]. Third, the study-level nature of the analysis limits the ability to explore individual-level data and precludes causal inferences. Additionally, while GA emerged as a significant modifier, the underlying mechanisms and precise role of GA in the observed association require further investigation. Another limitation is the variation in diagnostic criteria for GDM across the included studies. Most studies applied the International Association of the Diabetes and Pregnancy Study Groups (IADPSG) criteria [22–24, 26–28, 36, 37], while one study used the Diabetes in Pregnancy Study Group India (DIPSI) criteria [25]. Differences in diagnostic thresholds may introduce variability in GDM classification, potentially affecting the pooled estimates [59]. However, due to the limited number of included studies, we were unable to assess the impact of these variations. Future meta-analyses with a larger number of studies could allow for a more detailed evaluation of how different GDM diagnostic criteria influence fEFT. Lastly, the included studies used different ultrasound techniques for fEFT measurement, contributing to heterogeneity in our meta-analysis. The absence of a standardized protocol for fEFT assessment underscores the need for future studies to establish uniform measurement criteria, improving comparability across studies. The findings of this meta-analysis have important clinical implications. Our results suggest that increased fEFT may serve as an early indicator of fetal metabolic risk in pregnancies complicated by diabetes. Given its non-invasive nature, ultrasound-based fEFT assessment could be integrated into prenatal screening protocols to identify fetuses at risk of metabolic complications [60]. Moreover, optimizing maternal glycemic control may help mitigate excessive fetal fat accumulation, potentially improving offspring’s long-term cardiometabolic health [12]. Future studies should establish standardized thresholds for fEFT measurement and assess its predictive value in clinical practice. Routine fEFT assessment in pregnancies complicated by DM could provide valuable information for risk stratification and guide targeted interventions to optimize fetal outcomes [61]. The identification of GA as a key modifier highlights the need for close monitoring during later gestation, particularly in women with poor glycemic control. While increased fEFT has been suggested as a marker of fetal metabolic compromise, including macrosomia [62], we were unable to assess its direct relationship with these outcomes due to limited available data. Future studies should investigate whether fEFT can serve as an early predictor of fetal overgrowth and metabolic dysfunction, particularly in pregnancies complicated by diabetes. Further research should focus on elucidating the long-term implications of increased fEFT on offspring health and exploring interventions to mitigate these effects [63]. Longitudinal studies tracking fEFT from fetal to postnatal life and its association with offspring cardiometabolic outcomes would be particularly informative [63]. Additionally, randomized controlled trials evaluating the effects of glycemic control and other maternal interventions on fEFT and offspring health could provide critical insights into causal pathways and potential prevention strategies.

## Conclusion

In conclusion, this meta-analysis demonstrates that maternal DM during pregnancy is associated with increased fEFT, with the effect becoming more pronounced in later gestation. These findings underscore the importance of glycemic control and targeted monitoring in diabetic pregnancies to mitigate long-term cardiometabolic risks in offspring. Further research is needed to elucidate the mechanisms underlying this association and to assess the clinical utility of fEFT as a prognostic marker in this high-risk population.

## Supplemental data

**PubMed from inception to November 12, 2024**

**Table:** 

**#**	**Searches**	**Results**
**1**	“epicardial adipose tissue”[MeSH] OR “epicardial adipose tissue” OR “epicardial fat” OR “pericardial adipose tissue” OR “pericardial fat” OR “cardiac adipose tissue” OR “cardiac fat” OR “subepicardial adipose tissue” OR “subepicardial fat” OR ”heart fat” OR “heart adipose tissue”	**1553**
**2**	(“gestational diabetes”[MeSH] OR “gestational diabetes” OR “GDM” OR “pregestational diabetes” OR ((“gestational” OR “pregnancy”[MeSH] OR “pregnant” OR “pregestational”) AND (“diabetes”[MeSH] OR “diabetic” OR “hyperglycemia”[MeSH])))	**3736**
**3**	1 and 2	**88**

**Embase from inception to November 12, 2024**

**Table:** 

**#**	**Searches**	**Results**
**1**	“epicardial adipose tissue”/exp OR “epicardial adipose tissue” OR “epicardial fat” OR “pericardial adipose tissue” OR “pericardial fat” OR “cardiac adipose tissue” OR “cardiac fat” OR “subepicardial adipose tissue” OR “subepicardial fat” OR “heart fat” OR “heart adipose tissue”	**7542**
**2**	“gestational diabetes mellitus”/exp OR “gestational diabetes” OR “GDM” OR “pregestational diabetes” OR (“gestational” OR “pregnancy”/exp OR “pregnant” OR “pregestational”) AND (“diabetes”/exp OR “diabetic” OR “hyperglycemia”/exp)	**31262**
**3**	1 and 2	**68**
**4**	Limit 3 to human	**62**
**5**	Limit 4 to clinical study	**60**
**6**	Limit 5 to Embase	**52**

**Web of Science from inception to November 12, 2024**

**Table:** 

**#**	**Searches**	**Results**
**1**	TS ═ “epicardial adipose tissue” OR “epicardial fat” OR “pericardial adipose tissue” OR “pericardial fat” OR “cardiac adipose tissue” OR “cardiac fat” OR “subepicardial adipose tissue” OR “subepicardial fat” OR “heart fat” OR “heart adipose tissue”	**5714**
**2**	TS ═ “gestational diabetes” OR “GDM” OR “pregestational diabetes” OR (“gestational” OR “pregnancy” OR “pregnant” OR “pregestational”) AND (“diabetes” OR “diabetic” OR “hyperglycemia”)	**11312**
**3**	1 and 2	**28**

## Data Availability

The data that support the findings of this study are available from the corresponding author upon reasonable request.
